# Novel protocol to establish the myocardial infarction model in rats using a combination of medetomidine-midazolam-butorphanol (MMB) and atipamezole

**DOI:** 10.3389/fvets.2022.1064836

**Published:** 2022-12-05

**Authors:** Ahmed Farag, Ahmed S. Mandour, Lina Hamabe, Tomohiko Yoshida, Kazumi Shimada, Ryou Tanaka

**Affiliations:** ^1^Department of Veterinary Surgery, Faculty of Veterinary Medicine, Tokyo University of Agriculture and Technology, Fuchu, Japan; ^2^Department of Surgery, Anesthesiology, and Radiology, Faculty of Veterinary Medicine, Zagazig University, Zagazig, Egypt; ^3^Department of Animal Medicine (Internal Medicine), Faculty of Veterinary Medicine, Suez Canal University, Ismailia, Egypt

**Keywords:** myocardial infarction, rat, MMB, atipamezole, anesthesia, ECG, echocardiography

## Abstract

**Background:**

Myocardial infarction (MI) is one of the most common cardiac problems causing deaths in humans. Previously validated anesthetic agents used in MI model establishment are currently controversial with severe restrictions because of ethical concerns. The combination between medetomidine, midazolam, and butorphanol (MMB) is commonly used in different animal models. The possibility of MMB combination to establish the MI model in rats did not study yet which is difficult because of severe respiratory depression and delayed recovery post-surgery, resulting in significant deaths. Atipamezole is used to counter the cardiopulmonary suppressive effect of MMB.

**Objectives:**

The aim of the present study is to establish MI model in rats using a novel anesthetic combination between MMB and Atipamezole.

**Materials and methods:**

Twenty-five Sprague Dawley (SD) rats were included. Rats were prepared for induction of the Myocardial infarction (MI) model through thoracotomy. Anesthesia was initially induced with a mixture of MMB (0.3/5.0/5.0 mg/kg/SC), respectively. After endotracheal intubation, rats were maintained with isoflurane 1% which gradually reduced after chest closing. MI was induced through the left anterior descending (LAD) artery ligation technique. Atipamezole was administered after finishing all surgical procedures at a dose rate of 1.0 mg/kg/SC. Cardiac function parameters were evaluated using ECG (before and after atipamezole administration) and transthoracic echocardiography (before and 1 month after MI induction) to confirm the successful model. The induction time, operation time, and recovery time were calculated. The success rate of the MI model was also calculated.

**Results:**

MI was successfully established with the mentioned anesthetic protocol through the LAD ligation technique and confirmed through changes in ECG and echocardiographic parameters after MI. ECG data was improved after atipamezole administration through a significant increase in heart rate (HR), PR Interval, QRS Interval, and QT correction (QTc) and a significant reduction in RR Interval. Atipamezole enables rats to recover voluntary respiratory movement (VRM), wakefulness, movement, and posture within a very short time after administration. Echocardiographic ally, MI rats showed a significant decrease in the left ventricular wall thickness, EF, FS, and increased left ventricular diastolic and systolic internal diameter. In addition, induction time (3.440 ± 1.044), operation time (29.40 ± 3.663), partial recovery time (10.84 ± 3.313), and complete recovery time (12.36 ± 4.847) were relatively short. Moreover, the success rate of the anesthetic protocol was 100%, and all rats were maintained for 1 month after surgery with a survival rate of 88%.

**Conclusion:**

Our protocol produced a more easy anesthetic effect and time-saving procedures with a highly successful rate in MI rats. Subcutaneous injection of Atipamezole efficiently counters the cardiopulmonary side effect of MMB which is necessary for rapid recovery and subsequently enhancing the survival rate during the creation of the MI model in rats.

## Introduction

Myocardial infarction (MI) is the main form of ischemic heart disease which occurs due to the blockage of one or more coronary vessels leading to myocardial ischemia and necrosis (1). Acute MI remains the most severe form of coronary artery disease in humans, accounting for almost 4 million deaths each year in Europe and Northern Asia (2). The development of a simple, effective, and time-saving MI animal model is crucial to enrich our knowledge about its pathophysiology and finding better therapeutic options. Various methods have been established in laboratory animal models (3), either by using chemicals that interrupt the coronary circulation (4), coronary artery ligation *via* open thoracic cage surgery (5), or through non-invasive catheter method (6).

Owing to an increasing concern for laboratory animal welfare and third-party certification of experimental facilities, advances in rodent anesthesia are currently a topic of interest. In MI models, achieving an appropriate anesthetic effect is not only essential from the welfare viewpoint but also constitutes a great challenge that controls the success rate of the model. Generally, achieving an appropriate anesthetic effect in rodent MI models requires sufficient anesthetic depth and fewer cardiorespiratory depression. It has reported that cardiorespiratory depression affecting recovery and survival rate post-MI, subsequently affecting experimental data and the statistical power (7). As a result, an anesthetic protocol that mediates appropriate anesthetic depth while minimizing cardiorespiratory impact is worth investigating, suitable for real-time control of anesthetic depth, and might be used for both short or long durations (8).

In veterinary studies, various anesthetic combinations have been previously proven to be effective to induce MI models in rodents such as ketamine and xylazine (9, 10), a mixture of ketamine, xylazine and acepromazine (11), sodium pentobarbital (12), chloral hydrate 10% (13, 14) and inhalational anesthesia as isoflurane (induction: 5%, maintenance: 2.5%) with buprenorphine was subcutaneously administered for analgesia (15, 16).

For instance, Isoflurane has the advantages of rapid induction of anesthesia, rapid recovery, and minimal influence on hepatic metabolism; however, isoflurane may produce some unfavorable respiratory depression (7), and is not sufficient to provide adequate analgesic effect when highly invasive surgical procedures are needed (17, 18). Although ketamine is widely used in MI model induction (19), ketamine is currently classified as a narcotic drug and many countries have strengthened restrictions on its purchase, storage, and associated record-keeping procedures (20). Moreover, pentobarbital sodium is inappropriate as a general anesthetic due to its minimal analgesic effect and narrow safety margin with the undesirable cardiodepressive effects of reducing heart rate, stroke index, and cardiac index in rodents (19, 20). Consequently, the ethical restrictions and limitations of some medicines which are crucial in MI model induction increase the challenges for successful MI model establishment.

The combination between medetomidine, midazolam, and butorphanol (MMB) anesthetic is recently used in experimental animal studies as a substitute for ketamine or pentobarbital sodium (21). The anesthetic duration of MMB is longer than those of ketamine or pentobarbital sodium and is often associated with depression of respiration and circulation, and reduction of general motor activity and neuronal activity (21). These side effects may limit the usability of MMB in MI model induction. More specifically, medetomidine causes significant cardio-respiratory depression (22). Atipamezole, a synthetic α2-adrenergic antagonist, can antagonize medetomidine-induced respiratory depression and results in rapid recovery from MMB anesthesia (21). Atipamezole is also effective for reducing the maintenance concentration of isoflurane which leads to the amelioration of cardio-respiratory depression induced by isoflurane (17).

To our knowledge combination between MMB and atipamezole has never been studied in MI models. The objective of the study is to provide an easy and successful protocol to induce the MI model using MMB, and atipamezole. Our result will be helpful for an easily induced model using ethically approved medications with a higher success rate for research purposes.

## Materials and methods

### Animals and ethical approval

The study was conducted on 25 male Sprague Dawley (SD) rats, 12 to 15 weeks of age, and weighing between 350 and 400 gm. All procedures followed the Guide for the Care and Use of Laboratory Animals and were approved by the Institutional Animal Care and Use Committee of the Tokyo University of Agriculture and Technology (Approval No R04-185). The rats had free access to food and water and were housed at 25°C with a 12 h light/dark cycle.

### Anesthetic agents

The following anesthetics were used: Medetomidine hydrochloride (Domitor^®^, Orion Pharma Animal Health, Helsinki, Finland), Midazolam (Dormicum^®^, Astellas Pharma Inc., Tokyo, Japan), Butorphanol (Vetorphale, Meiji Seika Pharma Co., Ltd.), Isoflurane Inhalation Solution (Isoflurane, Pfizer Inc., New York, USA) and Atipamezole (ATI) (Antisedan, Orion Pharma Animal Health).

### Anesthesia protocol

Firstly, a mixture was prepared by mixing medetomidine hydrochloride, midazolam, and butorphanol (MMB) at a dose rate of 0.3, 5.0, and 5.0 mg/kg BW (23, 24). The anesthetic mixture was freshly prepared and diluted with sterile saline as stock solution as described in Table 1. Rats were subcutaneously injected at a dose rate of 0.5 ml of mixture/100 gm BW. Following the loss of front paw reflex, hind paw reflex, tail reflex, corneal reflex, and body righting reflex, rats were rapidly transferred to endotracheal intubation and maintained with isoflurane 1.0 % using a rodent inhalant anesthesia apparatus.

**Table 1:** Concentrations and doses of the used anesthetic agents.

**Agent**	**Concentration (mg/ml)**	**Dose (mg/kg)**	**Volume in saline (10 ml)**	**Administered volume (ml/kg)**
Medetomidine	1.0	0.3	0.3	0.3
Midazolam	5.0	5.0	1.0	1.0
Butorphanol	5.0	5.0	1.0	1.0
Atipamezole	5.0	1.0	2.0	1.0

### Induction of MI model

Following the above-mentioned anesthetic protocol, the animals were intratracheally intubated using a 16-gauge intravenous catheter before being placed in a supine position on a temperature-controlled pad at a core temperature of 35.5°C. A small incision between the third and fourth intercostal spaces was made to perform a left-sided thoracotomy. A blunt-ended retractor was used to expand the incision away from the lung to avoid its collapse. To access the heart, the pericardial sac was cut open and the site of coronary artery ligation was determined. The site of ligation of the left anterior descending (LAD) coronary artery was determined 8 mm away from the origin, then a 6-0 prolene ligature was passed underneath the LAD and secured with three knots using a tapered atraumatic needle (9). Successful ligation was confirmed by visible blanching and cyanosis of the anterior wall of the left ventricle, as well as swelling of the left atrium (25). Ribs and muscles were closed using 3-0 vicryl absorbable sutures leaving a small gap to aspirate air left in the thoracic cavity. The air was aspirated through a tube (2 mm in diameter) without touching the lungs. Nonabsorbable suture materials, such as silk 3-0, were used to suture the skin. The surgical site was dressed daily to prevent infection and to monitor for suture site dehiscence.

### Administration of atipamezole

**Figure 1:**
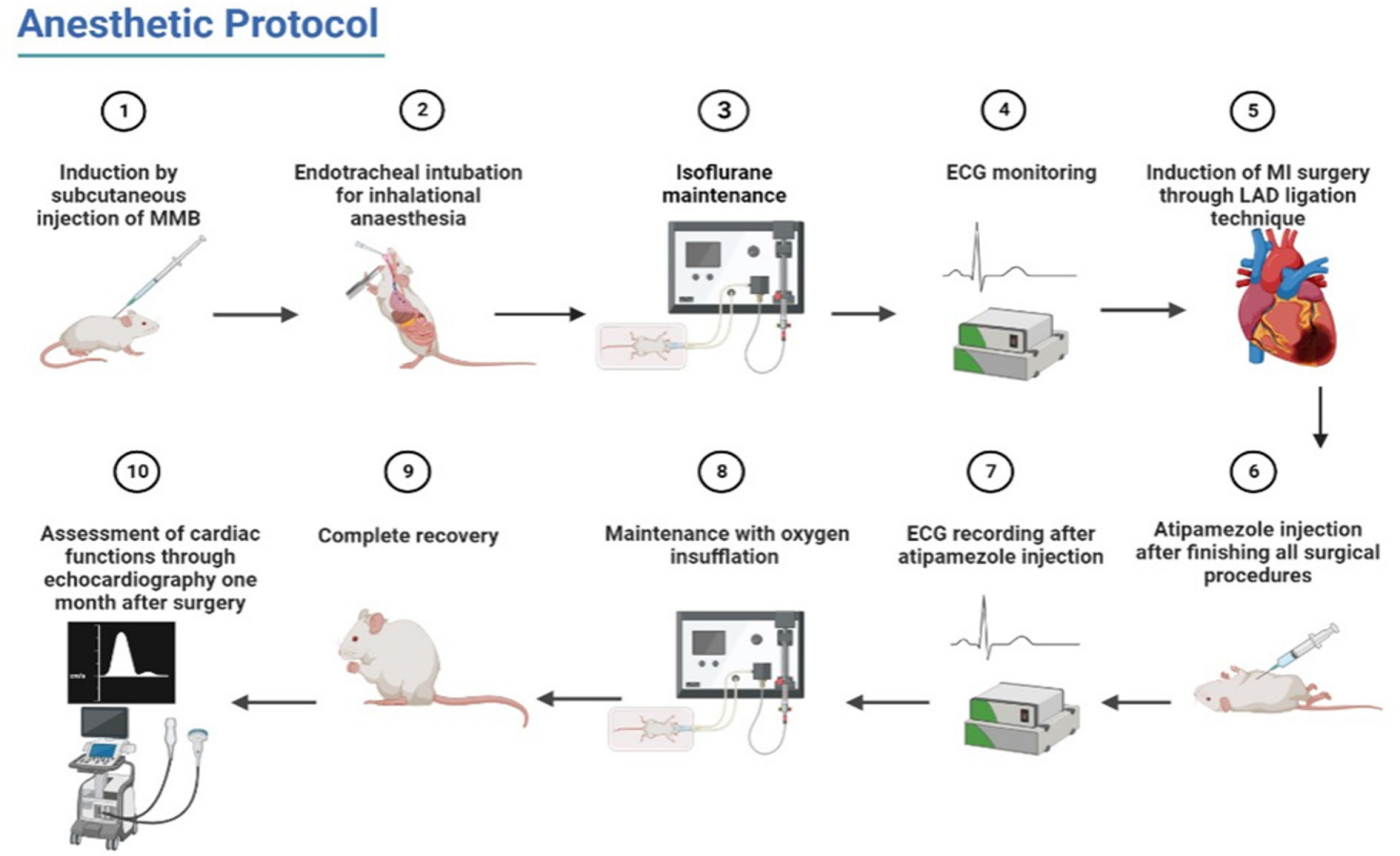
Schematic illustration of the used procedures of anesthetic protocol from induction to recovery and post-MI confirmation.

After LAD ligation and closing of the chest, the maintenance with isoflurane was reduced to 0.5% till suturing the skin and finishing the surgical procedures. At this point, atipamezole was injected subcutaneously at a dose rate of 1.0 mg/kg (Table 1). Then, isoflurane was stopped, and rats were maintained with oxygen insufflation (2 l of oxygen per minute). After observing the initial movement of rats, the endotracheal tube was removed slowly, and rats were changed to a nose cone mask (additional oxygen insufflation) till complete recovery (Figure 1).

### Post-operative care

Standard postoperative procedures were followed to control pain and infection. After recovery, all animals were treated with gentamicin (Nacalai Tesque Co., Ltd., Tokyo, Japan) which was injected intraperitoneally (2.4 × 10^4^/kg/day) for 3 days (14) while carprofen (Rimadyl^®^, Zoetis Japan K.K., Tokyo, Japan) was used with a pre-surgical dose of 5.0 mg/kg/SC, followed by two post-surgery injections (26).

### Measurement of time intervals during operation

Induction time was defined as the duration from injection of anesthetic mixture to the start of the loss of a body-righting reflex. Operation time was defined as the duration from the start of MI surgery till the end of all surgical procedures. Partial recovery time was defined as the duration from the end of the operation period (atipamezole injection) till trials of animals to remove the endotracheal tube (initial movement). Meanwhile, complete recovery time was defined as the duration from the removal of the endotracheal tube and the change to a nose cone mask to restore all vital reflexes. All these durations were recorded and expressed as mean ± SD.

### ECG monitoring

The ECG signals were recorded with needle electrodes connected with PowerLab hardware (ML880 PowerLab 16/30, AD Instruments) and LabChart Pro software (LabChart v8, AD Instruments) using a previously published protocol (27). The setting of PowerLab for ECG measurements followed the instructions provided by the producer. The ECG recording started 10 min after the animal was anesthetized with (MMB; i.e., before atipamezole injection) and then was conducted for 10 min and repeated after atipamezole administration for another 10 min. For each record, the most stable continuous segment was chosen for ECG analysis.

The ECG was recorded in an anesthetized state before and after atipamezole administration ECG parameters included RR Interval (s), heart rate (HR), atrial complex (PR interval, P wave duration, and P wave amplitude), ventricular complexes (QRS complex, QT and QTc interval duration) were recorded and analyzed.

### Confirmation of MI model

Cardiac functions were evaluated directly before and 1 month after MI. The echocardiographic machine (Hitachi-Aloka Medical Ltd., Tokyo, Japan, ProSound F75 ultrasonographic system) with a 12-MHz transducer and simultaneous ECG was used. The echocardiography was performed in accordance with the guidelines of the American Society of Echocardiography (ASE) (28, 29). All animals were anesthetized with MMB subcutaneously administered for easy and feasible examination and at the level of the papillary muscles, a two-dimensional right parasternal short-axis view of the LV was achieved using M-mode. LV was measured manually by the same observer using the ASE's leading-edge method (30), which has been validated for the rat MI model (31).

The LV internal diameter during diastole (LVIDd), LV internal diameter during systole (LVIDs), LV posterior wall diameter during diastole (LVPWd) systole (LVPWs) and (IVSd) and (IVSs) interventricular septal thickness in end-diastole and systole, respectively. Ejection fraction (EF%), and fractional shortening (FS%) were obtained from that view. From each rat, each echocardiographic parameter was measured five times and the data were averaged (32).

### Statistical analysis

Data analysis was performed using GraphPad Prism8 version 7.01 (GraphPad Software, Inc, San Diego, California). The normality of the data was tested by the Shapiro–Wilk test. To compare the cardiac function parameters before and after MI model induction, the student *T*-test was used and a *P* < 0.05 was considered statistically significant.

## Results

### Operation intervals and success of the MI model

Initially, rats were subcutaneously administered MMB at a dose rate of 0.5 ml/100 gm BW. Anesthetic induction was generally quick and easy, and most rats were orotracheally intubated in the range of 2–5 min as Mean ± SD (3.440 ± 1.044) after MMB administration. Muscle relaxation and analgesia were sufficient to begin thoracotomy immediately after anesthetic administration and the recovery time was divided into two stages: partial as Mean ± SD (10.84 ± 3.313 min) and complete as Mean ± SD (12.36 ± 4.847 min). The total operation time was 29.40 ± 3.663 min as Mean ± SD (Table 2). The success rate of the anesthetic protocol was 100%. In addition, all animals used in the current study were maintained for 1 month after surgery with a survival rate of 88% (22/25), and three rats died within 24 h after MI induction.

**Table 2:** Measurements of the anesthetic protocol durations.

**Measurement**	**Mean ±SD**
Induction time	3.440 ± 1.044
Operation time	29.40 ± 3.663
Partial recovery time	10.84 ± 3.313
Complete recovery time	12.36 ± 4.847

### Assessment of ECG

**Figure 2:**
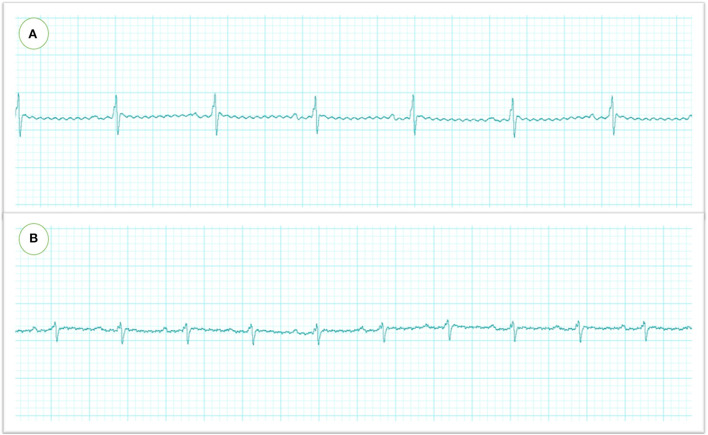
Electrocardiographic recordings in rats were measured by needle electrodes with Lab chart. The heart rate and RR intervals were significantly reduced after MMB injection (before atipamezole administration) **(A)** which were restored to the normal level after atipamezole administration **(B)**.

**Figure 3:**
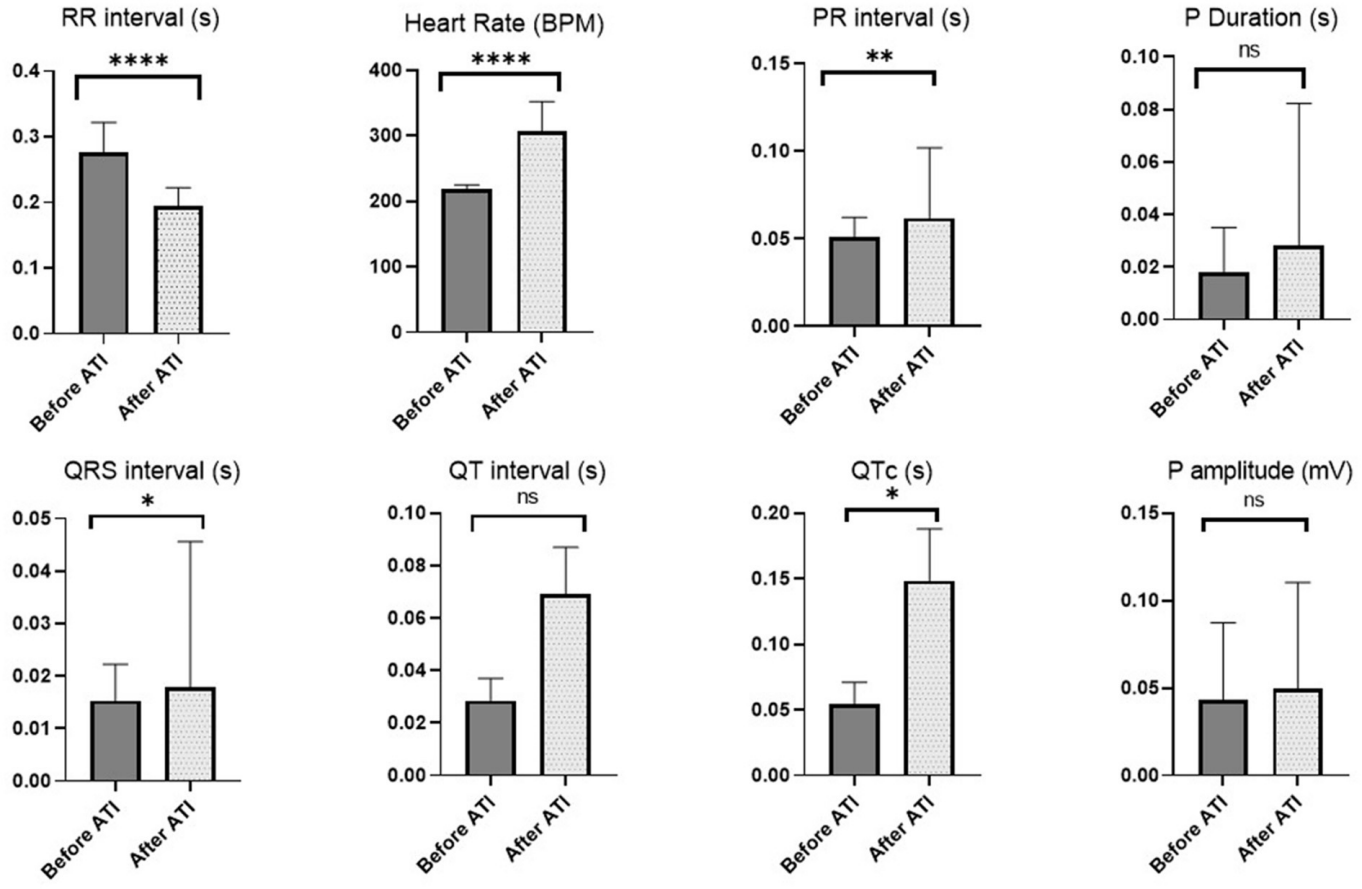
Changes in ECG parameters before atipamezole (i.e., directly after MMB) and after atipamezole administration. Asterisk used to indicate the significance, Ns *P* > 0.05 , *P ≤ 0.05, **P ≤ 0.01, ****P ≤ 0.0001.

The ECG analysis before and after atipamezole injection in the operated rats is illustrated in Figures 2, 3. There were no significant differences in P duration, P amplitude, and QT interval. However, the RR interval was significantly decreased after atipamezole administration (*P* = 0.0001). In contrast, other ECG parameters such as HR, PR Interval, QRS Interval, and QTc were significantly increased (*P* = 0.0001, 0.002, 0.043, and 0.018, respectively) when compared with their values before atipamezole injection.

### Confirmation of MI model

**Figure 4:**
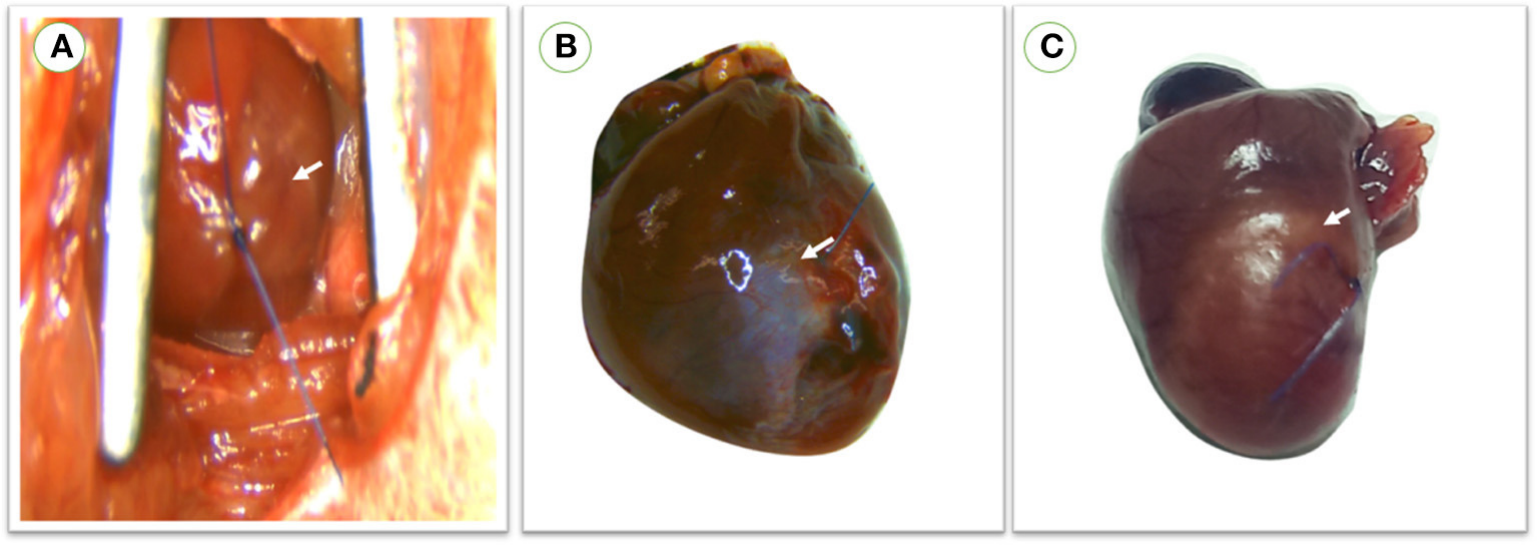
**(A)** During operation, myocardial infarction was confirmed in rats directly after LAD ligation through apparent blanching and cyanosis of the anterior wall of the left ventricle and swelling of the left atrium. **(B)** Color change of the left ventricular wall in survived rats which were maintained for 1 month with a small infarction size. **(C)** Large infarction size was observed in rats within 24 h following MI induction. red arrow; the site of infarction.

The myocardial infarction model was successfully created with the aforementioned anesthetic protocol *via* LAD ligation approach, as evidenced by apparent blanching and cyanosis of the anterior wall of the left ventricle and swelling of the left atrium immediately following artery ligation. No clinical abnormalities were observed during the observation period. Three rats died within 24 h post-MI induction and showed rapid respiration and off food (Figure 4).

**Figure 5:**
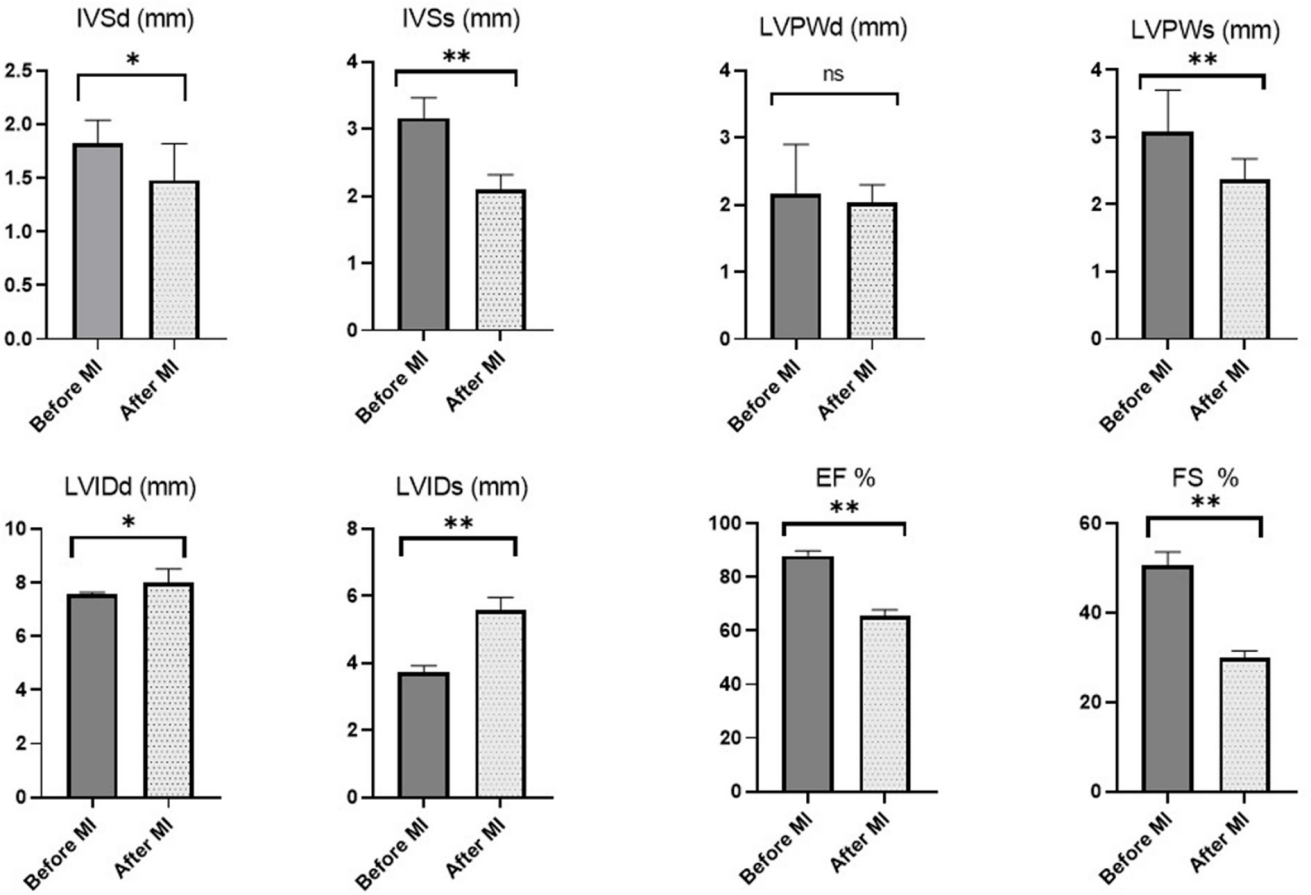
Echocardiographic measurements in rats before MI induction and 1 month later. Asterisk used to indicate the significance, Ns *P* > 0.05 , *p < 0.05, **p < 0.01.

**Figure 6:**
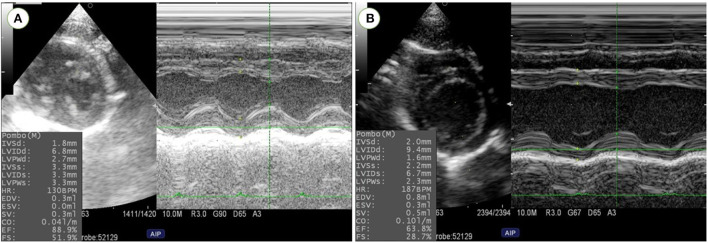
Left ventricular dimensions and function were evaluated using M-Mode echocardiography at right parasternal short axis in rats before MI induction **(A)** and one-month post-MI **(B)**. Reduction in left ventricular wall thickness and cardiac function and increase in left ventricular diameters were confirmed in MI model rats.

The echocardiographic parameters measured before and 1 month after MI induction are summarized in Figure 5. Changes in echocardiographic parameters can be seen after MI, as a significant decrease was observed in IVSd, IVSs, LVPWs, EF% and FS% (*P* = 0.047, 0.007, 0.007, 0.007, and 0.007, respectively). In addition, a significant increase was recorded in LVIDd and LVIDs after LAD ligation (*P* = 0.039; 0.007, respectively) (Figure 6).

## Discussion

The rat model is the most commonly used to study the pathophysiology of cardiovascular diseases including ischemic heart diseases as well as other models (33, 34). Limitations regarding the anesthetic protocols because of animal welfare, ethical concerns, and public health circumstances limit the usability of well-known medications in MI models, making the establishment of MI models more difficult. In addition, scientific publications still cannot be relied upon to present a detailed description of analgesia and anesthesia protocols. Most recently, an assessment of anesthetic and analgesic regimens in publications involving non-human primates revealed the absence of critical details reporting (35). In the present study, we have developed a successful MI induction in all rats with a survival rate of 88% using the novel protocol of anesthesia (combination of MMB + Isoflurane and countered with atipamezole). The success rate of the anesthetic protocol was 100% which provides quick and easy induction of general anesthesia with facilitating orotracheally intubated, muscle relaxation and analgesia were sufficient to perform the surgery recording a very short recovery time with minimal side effects in comparison to other previous studies. Our protocol may be introduced as an alternative to ketamine, xylazine, and pentobarbital with sufficient anesthetic and time-saving effects in rats.

Regarding the anesthetic combination, the used MMB mixture has been created based on previous reports in rats and mice (23, 24, 36–39).

Midazolam is a benzodiazepine that is water soluble. In rodents, pigs, and primates, benzodiazepines can cause significant sedation; however, they are not analgesic and do not create a true general anesthetic state (40). Midazolam is used in conjunction with other drugs to induce anesthesia (41). Medetomidine is a more potent imidazole derivative than xylazine, with higher alpha2-adrenoceptor selectivity (41). Butorphanol, a synthetic opioid agonist-antagonist, is used in veterinary medicine as an analgesic agent (42). The combination of medetomidine, midazolam, and butorphanol has been reported as a reliable and safe anesthetic agent in the dog (43), sea lions (44), and red fox (45). In our study, we administered MMB in rats by subcutaneous injection as it is considered a more effective, and induced rapid, complete, and stable anesthetic effect than intraperitoneal injection (23). The intraperitoneal delivery route is the most commonly used for MMB administration in rats (37), but according to Sorrenti et al. (39), the induction time for a single dose of MMB combination administered subcutaneously in Sprague-Dawley rats was approximately 10 min; however, with intraperitoneal injection, this duration increased to 25 min and required one or two additional doses (39).

Isoflurane is known to have a relatively strong respiratory depression in various species (17, 46). Therefore, the main purpose of the current novel anesthetic protocol was to attenuate cardio-respiratory depression by reduction of isoflurane concentration and minimizing MMB side effects, producing more safe and time-saving protocol for induction of MI in rats.

Atipamezole is a highly selective 2-adrenergic antagonist that is known to counteract the anesthetic effect of MMB. We administrated atipamezole with the prescribed dosage as the recovery from the anesthetic effect and hypothermia was dosage-dependent, and even after a low dose of atipamezole, the same as medetomidine, the sedation continued, even after all reflexes had been restored. As a result, giving atipamezole at the same dose as medetomidine is insufficient to promote recovery from MMB anesthesia. In addition, atipamezole's ability to counteract MMB-induced anesthesia is partially attributable to the fact that MMB primarily exerts its anesthetic effect *via* the 2-adrenoceptor (47). Furthermore, it has been reported that atipamezole can also counteract the anesthetic effect of the combination of medetomidine, butorphanol, alfaxalone, and neurosteroid anesthetic, as well as can counter the anesthetic effect of MMB (48).

Generally, medetomidine is helpful in central analgesia while butorphanol is necessary for visceral analgesia (49, 50). Administration of atipamezole during early surgical procedures will abolish the analgesic effect of medetomidine and exposes the rat to pain. To avoid such situation in the current study, atipamezole was administrated after finishing all surgical procedures. In other words, during the operation, anesthesia was achieved through the effect of the used combination with isoflurane, and postoperative analgesia was achieved successfully through pain killer.

Rats were kept under oxygen insufflation (2 l of oxygen per minute) when isoflurane maintenance was stopped to prevent problems like hypoxia. This is in agreement with Mechelinck et al., who claimed that rats under ketamine-xylazine anesthesia are susceptible to hypoxia. This could result in an increase in delayed mortality from hypoxia-related lung failure. So they recommend using additional oxygen insufflation with the prescribed dose (51). Moreover, Ballard and Spadafora (52) stated that respiratory depression caused by ketamine-xylazine narcosis seems to be the key factor in lung damage. In principle, rats' lungs can be damaged by hypoxemia. This damage begins 8 h after the hypoxic incident with pulmonary edema, most likely due to sympathetic activation, increased vascular permeability, and hypoxic pulmonary vasoconstriction, and is followed by inflammation, pulmonary fibrosis, and vascular hypertrophy (52).

In our study, we reported that the induction time of our anesthetic protocol was < 5 min, these results were resemble that recorded with the ketamine/xylazine protocol (20), but with a short recovery time in total when compared to other protocols including ketamine/xylazine (20), MMB alone (23) and Pentobarbital (35). In other studies, The mean recovery time without atipamezole injection was 44.5 min and 50.0 min in males and females respectively (53).

All the operated rats in the present study were maintained for 1 month with a survival rate of 88%, and three rats died within 24 h post-surgery due to surgical errors, Lindsey et al. (54) stated that perioperative death within 24 h post-MI is usually due to surgical errors (or very large infarct sizes), and in the permanent occlusion MI model in mice, postoperative death may be due to rupture, acute heart failure, or arrhythmias (54).

The normal heart rate of rats has been reported 330–480 beats per minute (55). Kirihara et al. (56) stated that MMB had decreased heart rate and blood oxygen saturation in rats (56). The recorded heart rate in our study was in the range of 187 to 226 beats per minute, confirming the bradycardia expected with the use of an α2-agonist (57). After the administration of atipamezole, heart rate began to increase within 2 min and was fully restored after 4 min to reach 270–352 beats per minute. According to ECG data from our study, the main advantage of administering atipamezole is that it allows rats to recover voluntary respiratory movement, wakefulness, movement, and posture within a relatively short time after injection, this allows for faster extubating of operated rats, minimizing the post-anesthesia recovery period and, as a result, lowering the risk of side effects and residual effects of the anesthetics (58).

In operated rats, we found typical ischemic changes on transthoracic echocardiography, particularly significant increase in left ventricular diameters and a significant decrease in wall dimensions, EF%, and FS% following post-infarction LV remodeling in adult rats. Our findings are comparable to those of earlier studies on rats indicating a successful procedure (59, 60). Changes in cardiac function parameters with no clinical symptoms in the remaining rats suggest subclinical heart failure.

At 1-month post-infarction, morphological alterations such as an increase in LVESD and LVEDD, as well as functional changes such as a decrease in FS and EF, were clearly identified. Our data are similar to those of other reports (61–64). LAD-ligation significantly reduced regional contractility not just in the anterior, anteroseptal, and septal segments. Even adjacent regions like the lateral and posterior segments were affected. This can be explained by the distinctions between the geometries of the coronary arteries in rats and humans as there is no true circumflex artery and the LAD predominates in rats (65). As a result, the posterior and lateral regions of the left ventricle have a significant perfusion deficiency as a consequence of LAD ligation (64, 66). Differences in age, weight and echocardiographic transducers or procedures appear to provide slightly variable results in different laboratories. Based on our findings, we may conclude that we were successful in creating a novel anesthetic protocol for producing a MI model in adult rats and confirmed the efficacy of LAD ligation surgery under the used protocol.

Various animal models such as rats, rabbits, pigs and non-human primates, where sparse collateral coronary circulation is excisting, have been introduced to study MI; however, considerable mortalities due to delayed recovery is still controversial (67–69). Until now, our protocol did not utilized in any animal model. Therefore, the current protocol worth studying in other MI or ischemic dysfunction models.

### Limitations

In our study, we did not compare our protocol to others such as ketamine and pentobarbital sodium. These drugs are no longer available in Japan and their import or use is not ethical due to being classified as narcotic drugs (20). In the current study, the effect of the used protocol on pulmonary function and blood pressure was not investigated. However, other studies have examined the detailed hemodynamic and respiratory impact of MMB alone or after antaonizing with atipamezole in rats and rabitts (23, 70) with no surgical approaches.

## Conclusion

To our knowledge, this is the first study to use anesthetic combination of MMB and a light concentration of isoflurane with atipamezole in MI rats. Subcutaneous injection of atipamezole efficiently counters the cardiopulmonary side effect of MMB which is necessary for rapid recovery and subsequently enhancing the survival rate during the establishment of the MI model. Our approach produced a more easily anesthetic effect and time-saving procedures with a highly successful rate in MI rats which may be effective in the research field of cardiothoracic disorders using rat models. Our protocol worth studying in other animal models as well.

## Data availability statement

The raw data supporting the conclusions of this article will be made available by the authors, without undue reservation.

## Ethics statement

The animal study was reviewed and approved by the experimental procedures were approved by the local Ethical Committee of the Tokyo University of Agriculture and Technology, Japan (Approval No R04-185).

## Author contributions

Experiment design: AF, AM, and RT. Induction of model: AF. Echocardiography and electrocardiography, data collection, and statistical analysis: AF and AM. Investigation: AF, AM, and KS. Writing and drafting: AF, TY, LH, and AM. Critical editing: AM. Supervision: RT. All authors reviewed and edited the final version.

## Conflict of interest

The authors declare that the research was conducted in the absence of any commercial or financial relationships that could be construed as a potential conflict of interest.

## Publisher's note

All claims expressed in this article are solely those of the authors and do not necessarily represent those of their affiliated organizations, or those of the publisher, the editors and the reviewers. Any product that may be evaluated in this article, or claim that may be made by its manufacturer, is not guaranteed or endorsed by the publisher.
